# The role of teachers' socio-emotional competence in reducing burnout through increased work engagement

**DOI:** 10.3389/fpsyg.2023.1295365

**Published:** 2023-10-31

**Authors:** Veronica Ornaghi, Elisabetta Conte, Valeria Cavioni, Eleonora Farina, Alessandro Pepe

**Affiliations:** ^1^“R. Massa” Department of Human Sciences for Education, University of Milano-Bicocca, Milan, Italy; ^2^Department of Humanistic Studies, Literature, Cultural Heritage, Education Sciences, University of Foggia, Foggia, Italy

**Keywords:** teachers' burnout, perceived stress, emotional competence, work engagement, structural equation modeling

## Abstract

**Introduction:**

Research has highlighted the relevance of socio-emotional competence in buffering the harmful impacts of perceived stress on the psychological facets of the teaching profession. The purpose of this paper is to innovatively investigate the relationships between perceived stress, work engagement, and burnout in a single comprehensive model, when considering the potential role that socio-emotional competence plays in mitigating the adverse impact of perceived stress on burnout.

**Methods:**

A total of 276 Italian in-service teachers (mean age = 46.6 ± 9.9 years) completed quantitative self-report measures of perceived stress, socio-emotional competence, work engagement, and burnout. Data were analyzed by using a structural equation modeling (SEM) approach.

**Results:**

All fit indexes supported the model's full acceptance and suggested that teachers' socio-emotional competence reduced the effect of perceived stress on the risk of burnout by increasing their level of work engagement.

**Discussion:**

The implications of the findings are discussed in terms of promoting interventions that target not only stress reduction but also foster teachers' socio-emotional competence in order to maintain a good level of work engagement and reduce the effect of stress on burnout.

## 1. Introduction

The profession of teaching is one that demands a significant amount of emotional labor and has been linked to high levels of stress and burnout among its practitioners. Perceived stress has been widely acknowledged as a strong predictor of burnout in the teaching profession. In turn, burnout can have negative implications, including decreased job satisfaction, lessened capacity to succeed at work, diminished self-esteem, and lower academic attainment, for both teachers and their students (Schaufeli et al., 2009; Khan et al., 2019). However, recent research has also highlighted the relevance of socio-emotional competence acting as a buffer against harmful impacts of perceived stress on the psychological facets of the profession (Jennings and Greenberg, 2009; Holeyannavar and Itagi, 2012). In this context, socio-emotional competence refers to a teacher's ability to understand and manage their own emotions and respond effectively to the emotions of others (Brackett et al., 2012; Nalipay et al., 2021). Teachers who have higher levels of socio-emotional competence appear to be better equipped to deal with stressful situations, while they continue to establish positive connections with students, and remain engaged in their work (Mérida-López et al., 2017).

The purpose of this paper is to explore the association between perceived stress, burnout, work engagement, and socio-emotional competence in the teaching profession. To be more specific, we will investigate the relationships between perceived stress, burnout and work engagement, as well as the potential role that socio-emotional competence plays in mitigating the adverse impact of perceived stress on professional burnout.

To begin, we prepared a summary of some existing studies on perceived stress and burnout in teaching, including elements that lead to these experiences as well as possible consequences for both in-service teachers and students. After that, we shifted our focus to socio-emotional competence, looking at the several ways in which it has been conceptualized as well as some research that links it to both teachers' perceived stress along with consequences on work engagement. We then investigated the cumulative patterns of association between perceived stress, socio-emotional competence, work engagement and burnout by mean of structural equation modeling. More specifically, we investigated the ways in which socio-emotional competence may help reduce the adverse impact of stress on professional burnout. The model also explored how perceived stress can hinder teachers' ability to remain engaged in their work, as well as the potential role of perceived stress on socio-emotional competence.

### 1.1. Perceived stress, burnout, and work engagement in the teaching profession

The teaching profession involves caring activities based on the teacher-student relationship and thus on the emotional, intellectual and personal growth of the student, but also on a considerable expenditure of physical and psychological energy on the part of the same teacher. Teachers have to cope with numerous stressors such as workload, role ambiguity, lack of support, classroom management difficulties, organizational problems, and so on (i.e., Mérida-López and Extremera, 2017). Therefore, a high risk of developing anxiety and stress as a consequence of being exposed to a wide range of work stressors in their daily activities has been shown (Cooper and Travers, 2012). In the face of the many actual stressors that characterize the teaching profession, what seems crucial is how teachers perceive and feel about their lack of control and unpredictability.

Perceived stress is an individual's subjective judgment of the factors that contribute to being stressed (Chang, 2009; Kyriacou, 2011). In particular, a person experiences high levels of stress when the demands of the situation are seen as being excessive as compared to the available resources and coping skills. How teachers perceive stress is significantly associated with the risk of burnout (Bottiani et al., 2019), which is defined as a prolonged response of a person chronically exposed to emotional and interpersonal stressors in the workplace (Maslach et al., 2001). Burnout includes three dimensions: emotional exhaustion, depersonalization or cynicism and reduced personal accomplishment (Maslach et al., 2001). Exhaustion occurs when a person feels that he or she has exceeded his or her physical and emotional limits, becomes unable to regain physical and mental energy, struggles to cope with a new task and to manage the people with whom he or she must deal to accomplish it. Cynicism occurs when the person begins to assume a detached and impersonal attitude at work, even with the people with whom he or she routinely works. This is a defense mechanism to protect oneself from exhaustion, which often results in an abandonment of one's values and ideals, a significant drop in motivation and an indifference toward performing any kind of task. Finally, the sense of ineffectiveness and lack of accomplishment is characterized by growing feelings of inadequacy as well as a loss of confidence in one's own abilities.

The consequences of experiencing burnout can be very severe. Research has shown that individuals who experience burnout tend to report feelings of being helpless, hopeless, and powerless (Schaufeli and Buunk, 2004). As such, these persons are more likely to develop symptoms of depression due to the consequences of the sustained and uncontrollable stress and emotional exhaustion experienced (Bianchi and Schonfeld, 2016; Capone et al., 2019). Interestingly, the study by Bianchi et al. (2014) conducted on over 5,575 teachers, found that 90% of those who displayed signs of job burnout also met the diagnostic criteria for depressive disorder. Hence, the prolonged difficulties of effectively managing one's environment and personal tasks to be able to actively tackle stressors is a key factor in established theories related to depression (e.g., Peterson et al., 1993).

The effect of perceived stress on burnout is amplified in difficult and challenging situations, as emerged world-wide during the COVID-19 pandemic (Lizana et al., 2021; Pellerone, 2021; Vargas Rubilar and Oros, 2021; Levante et al., 2023). In a recent study, Geraci et al. (2023) found that the pandemic emergency did indeed increase the risk of teachers' burnout and negatively impacted their level of work engagement, which is a persistent, positive and satisfying work-related mental state, characterized by vigor, absorption and dedication to work activities (Schaufeli et al., 2002).

The critical situation and increasingly challenging demands faced by teachers during the pandemic (lock-down, social distancing, use of remote instruction formats, etc.) veritably increased stress factors, levels of anxiety, and the incidence of burnout, which was found to be related to the prevalence of negative emotions and emotional dysregulation (Carroll et al., 2022).

### 1.2. The protective role of teachers' socio-emotional competence

Numerous studies have pointed out that social and emotional skills are powerful resources that help teachers manage negative feelings and face stressful and challenging situations (Brackett et al., 2010; Jennings, 2011; Mérida-López and Extremera, 2017; Cece et al., 2022; Cefai et al., 2022; Cavioni et al., 2023). Teachers' high emotional competence may increase their ability to cope with symptoms of burnout by reducing the experience of stress (Rey et al., 2016; Lester et al., 2020) and is strongly related to high levels of work engagement and job satisfaction, as well as to low levels of burnout (D'Amico et al., 2020). If teachers are able to regulate their emotions and make decisions for themselves, they will feel committed to and engaged in improving their professional competence and carrying out voluntary actions in their workplaces and toward their colleagues. In contrast, emotionally poor teachers will often be more affected by burnout (Brackett et al., 2010).

Emotional skills, such as self-regulation, may have a protective role in preventing negative working experiences of teachers, especially during challenging situations, namely the sudden changes brought about by online teaching and the difficulties in keeping and building relationships with students during the COVID-19 pandemic (Messineo and Tosto, 2023). During lockdown, which Italy experienced first compared to other Western countries, teachers suddenly lacked social support, a key factor sustaining their mental health and wellbeing (i.e., Froehlich et al., 2022; Li et al., 2022). This caused increased stress, uncertainty, and bewilderment. Within this situation, possessing high emotional skills came in handy and allowed for cushioning the impact of perceived stress on teacher's mental health. In this regard, the negative effect of COVID-19 on teachers' work engagement and burnout was found to be associated with their level of emotional intelligence: high levels of emotional intelligence were related to greater work engagement and lower levels of burnout (Geraci et al., 2023).

Teachers' social and emotional competence can be an important factor in preventing burnout and fostering a positive attitude toward their work (Montgomery and Rupp, 2005; Mérida-López et al., 2022). The ability to control one's emotions, one's empathy, one's capacity to form positive connections, and ability to manage positive and negative conflict effectively are all examples of social and emotional competences. Teachers who have developed social and emotional skills are better able to cope with the effects of perceived stress and maintain a sustainable level of work engagement (Bakker et al., 2004; Bakker and Bal, 2010; D'Amico et al., 2020). As a result of having these skills, they are able to adequately manage the emotional and relational demands of the job, which in turn creates a constructive learning environment for the students (Skaalvik and Skaalvik, 2016). Moreover, emotional competence is an important antecedent of work engagement, as confirmed in a very recent study by Mérida-López et al. (2023).

### 1.3. The present study

The primary goal of this study, which is part of a broader research project on the promotion of teachers' professional wellbeing across Europe (https://teachingtobe.eu/), was to investigate the impact of perceived stress on teacher burnout after accounting for the role of socio-emotional competence and work engagement in a sample of Italian teachers. Very little research, to our knowledge, has been undertaken on the cumulative network of interactions among the variables under inquiry, with a particular focus on socio-emotional competence in teachers.

It is of utmost importance for teachers' professional growth and the prevention of negative phenomena like clinical issues (e.g., anxiety, depression) to examine the correlations between perceived stress, social-emotional skills, work engagement, and professional burnout. By gaining an understanding of the ways in which these factors interact with one another, it may be possible to devise useful techniques to enhance the quality of teaching, improve the working environment, and support the mental health of educators. It is possible to establish a pleasant learning environment by investing in teachers' social and emotional skills training, which would allow instructors to successfully manage stress, maintain sustainable work engagement, and prevent burnout. In addition, the adoption of organizational policies that create job engagement and promote stress management can be of essential importance in the process of developing a healthy and sustainable work environment for teachers. These efforts would not only be beneficial to the teachers themselves, but also to students, who could have teachers who are more motivated, engaged, and capable of providing them a great education.

Having this in mind, the following hypotheses were formulated: (H1) we expected perceived stress to have a statistically significant negative direct effect on socio-emotional competence and work engagement, and a statistically significant positive direct effect on burnout; (H2) we expected to find statistically significant negative direct effect of work engagement on professional burnout; (H3) we expected socio-emotional competence to have a significant direct positive effect on work engagement and a negative effect on burnout; (H4) finally, we expected to find significant effect of teachers' age on the risk of burnout.

## 2. Method

### 2.1. Participants

The sample was composed of 276 in-service Italian teachers. Females comprised 90% of participants, and their mean age was 46.6 ± 9.9 years. They were recruited in kindergartens and primary schools (50.3%), middle and high schools (49.7%), located in both urban and rural areas of Northern Italy. The teachers had an average of almost 17 years' teaching experience (*M* = 16.91 years, SD = 11.1). 90% of them were employed full time. Prior to participating in the study, all individuals were told about the research objectives and signed an informed consent release form.

The inclusion criteria were as follows: (1) working as an in-service teacher and (2) agreeing to the terms of participation in the study. There were no exclusion criteria. We gathered a convenience sample using a non-probability sampling technique in which participants were chosen from the general community solely because they agreed to participate (Emerson, 2015). The study was carried out in compliance with the American Psychological Association's ethical principles and code of conduct and received the approval of the University of Milano-Bicocca Ethics Committee (protocol number: 0129650/21). Participants were free to withdraw from the study at any time and received no monetary or other financial reward. There were no conflicts of interest among the authors in relation to the research.

### 2.2. Measures and procedure

In order to assess teachers' socio-emotional competence, work engagement, perceived stress and burnout, participants were asked to complete the following self-report measures.

The Social and Emotional Competence of Teachers questionnaire (SECTRS; Tom, 2012), which consists of 25 items that measure four areas: teacher-student relationships (interactions between teachers and students), emotion regulation (teachers' ability to manage their emotions in challenging situations), social awareness (teachers' sensitivity to diversity and awareness how their practice impacts students), and interpersonal relationships (teachers' relationships with parents and school staff). Teachers were asked to express their agreement or disagreement with the items on a 6-point Likert scale, from 1 (strongly disagree) to 6 (strongly agree). In the original instrument, Cronbach's alpha coefficients for the four subscales ranged between 0.69 and 0.81 (Tom, 2012). In the current study, the four subscales' reliability coefficients ranged between 0.73 and 0.80.

The 9-item Bergen Burnout Inventory (BBI-9; Salmela-Aro et al., 2011) assesses teachers' level of burnout through three core dimensions: emotional exhaustion (three items, sample item: “I am snowed under with work”), cynicism (three items, sample item: “I feel that I have gradually less left to give”), and inadequacy (three items, sample item: “I frequently question the value of my work”). For each item, respondents were asked to express their degree of agreement on a 6-point Likert scale ranging from 1 = completely disagree to 6 = completely agree. For the present study, reliability (Cronbach's α) of the three scales were: emotional exhaustion (0.72), cynicism (0.83), and inadequacy (0.76). The overall reliability of the full set of items was equal to 0.88.

The Utrecht Work Engagement—Short Form questionnaire (UWE-SF; Schaufeli et al., 2006), also known as the Utrecht Work Engagement Scale, is a widely used psychological instrument for measuring work engagement. The short form consists of nine statements that participants are asked to score on a 7-point Likert-type scale ranging from 0 (never) to 6 (always). The statements are intended to assess how frequently a person has felt and thought about work in the previous week. The measurement model estimated three aspects of workplace engagement: vigor (having a lot of energy and mental resilience when working), devotion (being deeply immersed in one's job and having a sense of purpose), and absorption (being completely focused and happily engrossed in one's work). The Italian version of the UWE was adopted (Pisanti et al., 2008). For this study, the reliability measures (Cronbach's α) of the scales were: vigor (0.82), dedication (0.89), and absorption (0.72).

The Perceived Stress Scale (PSS; Cohen and Williamson, 1988) is a well-known psychological instrument that was developed with the intention of determining the degree to which individuals view their own lives as being stressful (Tavolacci et al., 2013). The scale is comprised of 10 items, each of which is a statement that the participant is asked to rate on a Likert-type response scale from 0 (never true) to 4 (very often true). The purpose of the statements is to determine the degree to which a person has experienced specific sensations and ideas associated with stress. For this study reliability of the overall scale was Cronbach's α = 0.82.

Finally, socio-demographic data were also collected in terms of age, gender, and years of teaching experience. Data were collected in October and November 2022. Before completing the online questionnaires, during a face-to-face meeting, teachers were informed about the research and were asked to provide their consent to participate in the study.

### 2.3. Strategy of data analysis

**Figure 1:**
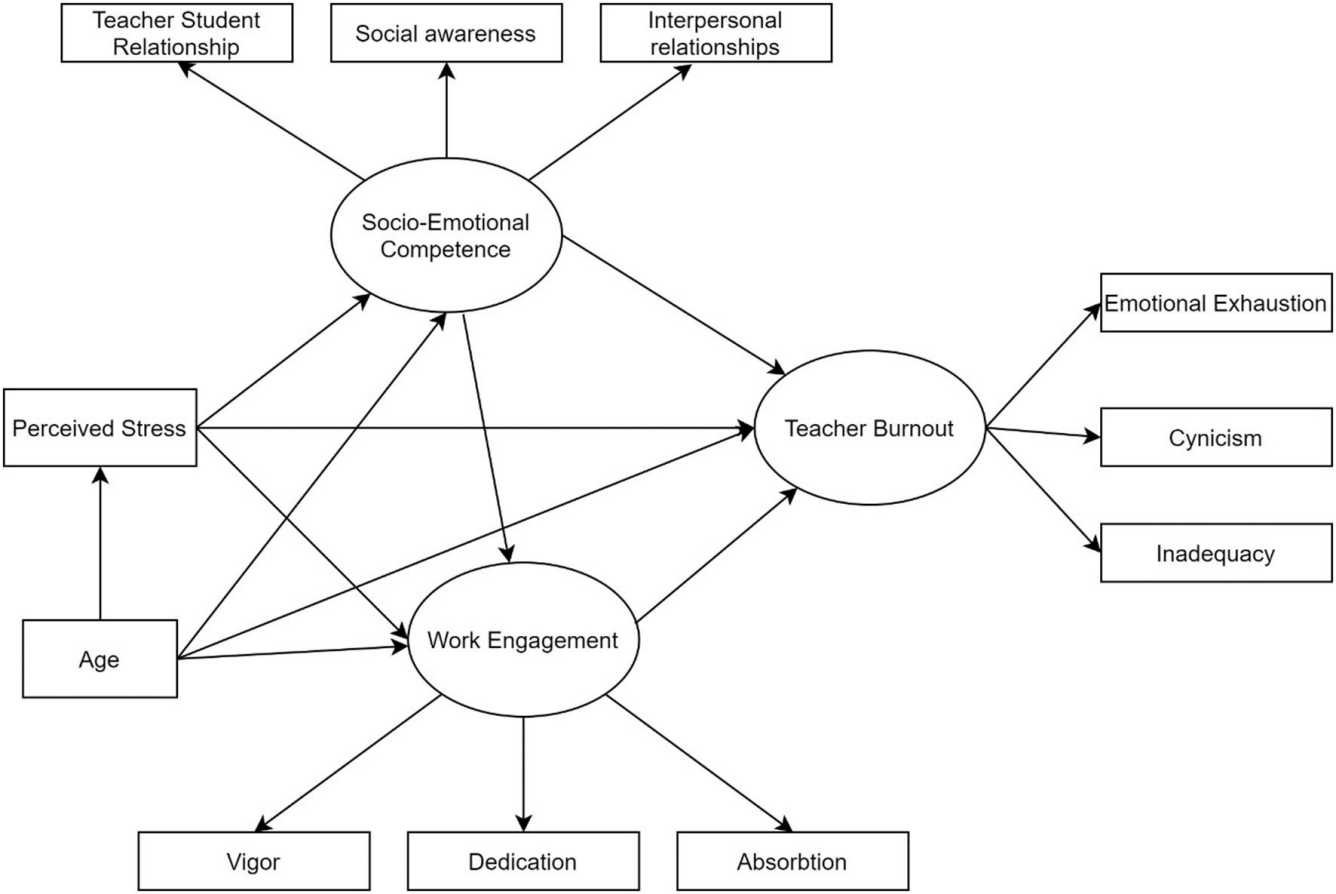
Conceptual model of perceived stress, teacher burnout, work engagement and socio-emotional competence.

In order to evaluate our conceptual model of perceived stress, socio-emotional competence, work engagement and teacher burnout, we adopted a structural equation modeling (SEM) approach. According to Preacher et al. (2011), the SEM approach has several advantages over other statistical approaches, such as multiple regression and analysis of variance, because it enables the simultaneous investigation of a large number of variables, it also takes into account the ways in which these variables are related to one another, and it evaluates the impact of latent variables on the way in which observed variables are related to one another. The SEM approach was utilized for the purpose of analyzing the relationships (both direct and indirect) that existed between the variables being studied. The SEM model used a series of mathematical equations to depict the direction and the strength of paths existing between observable and latent variables (i.e., factors that cannot be directly seen but can be inferred by the use of other measures) (Marcoulides and Schumacker, 2001; Thakkar, 2020). A SEM model requires testing the strength and direction of correlations between variables, examining the model's goodness of fit, and comparing alternative models to identify the one that provides the most accurate representation of the data in a wide variety of research topics, including psychology, sociology, medicine, and economics (Fiorilli et al., 2017; Conte et al., 2019; Ornaghi et al., 2019; Pepe et al., 2023). We opted to test this conceptual model using three endogenous latent variables and 10 empirical indicators (see Figure 1).

Starting from left to right, perceived stress was modeled as an exogenous variable. The model was then adjusted to include socio-emotional competence as a latent endogenous component assessed by the teacher-student interaction, social awareness, and interpersonal relationships. The emotion regulation sub-scale was not included in the measurement model due to collinearity issues with emotional exhaustion (e.g., VIF score over the suggested values for acceptance). Work engagement was similarly measured using three empirical indicators: vigor, devotion, and absorption. Finally, emotional exhaustion, cynicism, and inadequacy were used to quantify teacher burnout.

First, we investigated whether or not the data had a normal distribution for the normality score. None of the score sets had kurtosis or skewness values that were outside of the suggested ranges, which are 2–4 for kurtosis and −1 to 1 for skewness. After that, the data were examined using Mahalanobis distances with the intention of detecting multivariate outliers. The dataset did not require any cases to be eliminated because there were none that fit the elimination criteria. In the second stage, descriptive statistics and zero-order correlations were determined to evaluate preliminary associations between the variables that were the subject of the inquiry. In the end, a SEM model estimation was carried out using the Maximum Likelihood approach (Ripplinger and Sullivan, 2008). In accordance with standard SEM procedures, we computed confidence limits using a set of 500 random samples and a Monte Carlo simulation as well as the bootstrapping approach (Thakkar, 2020). We computed the stated indirect effects for each of the *k* examples, as well as the mean value based on all of the samples pooled together.

Five indices of goodness of fit were calculated to estimate model fit. These included (a) the Root Mean Square Error of Approximation (RMSEA), (b) the Standardized Root Mean Square Residual (SRMR), (c) the Goodness of Fit Index (GFI) (d) the Tucker-Lewis Index (TLI, TLI >0.95), and (e) the Comparative Fit Index (CFI). For the purposes of this study, the model was considered to fit the data if the RMSEA was <0.07 (Kenny et al., 2015), the SRMR was <0.05 (Ximénez et al., 2022), the NFI and TLI were >0.95 (Shi and Maydeu-Olivares, 2020), and the CFI was >0.95 (Marsh et al., 2014). All analyses were conducted using Statistical Package for Social Science (SPSS) version 21 and AMOS version 21.

## 3. Results

The following outline constitutes the structure of the results section of this paper. The first section presents the primary descriptive statistics as well as the pattern of zero-order correlations between the variables under study. Then, the findings of the structural equation model are reported, along with a comprehensive explanation of the direct and indirect effects that regulate the cumulative network of relationships between perceived stress, socio-emotional competence, work engagement, and burnout.

### 3.1. Descriptives and zero-order correlations

Table 1 summarizes the main statistical descriptives and zero-order correlations. Correlation analysis conducted on the cumulative scores obtained from the sum of the subscales of the different variables, showed that perceived stress was positively correlated with teacher burnout (*r* = 0.581), while it was negatively correlated with work engagement (*r* = −0.366) and socio-emotional competence (*r* = −0.313). In line with this result, burnout was also negatively correlated with work engagement (*r* = −0.517) and social-emotional competence (*r* = −0.276). Finally, the correlation between social-emotional competencies and work engagement was positive and statistically significant (*r* = 0.404). With regard to the sociodemographic variables, the gender of teachers did not appear to be correlated with any of the variables under investigation while the variable age showed a statistically significant and positive correlation with burnout (*r* = 0.248) while it did show a negative and statistically significant correlation with both work engagement (*r* = −0.167) and socio-emotional competence (*r* = −142). The set of correlations provided support for the use of the structural equation model by including only the age variable as a possible source of covariation to be estimated through the model.

**Table 1:** Overview of stress, teacher burnout, socio-emotional competence and work engagement: zero-order correlations and descriptives (*N* = 276).

	**1**	**2**	**3**	**4**	**5**	**6**
1. Gender	–					
2. Age	0.077	–				
3. Perceived Stress	0.052	0.049	–			
4. Teacher Burnout	0.013	0.248^**^	0.581^**^	–		
5. Work Engagement	0.099	−0.167^**^	−0.366^**^	−0.517^**^	–	
6. Socio-Emotional Competence	0.075	−0.142^*^	−0.313^**^	−0.276^**^	0.404^**^	–
Mean	–	46.55	17.29	22.31	44.85	117.34
Standard Deviation	–	9.94	6.34	9.16	6.81	12.51
Skewness	–	−0.382	0.051	0.763	−0.974	−0.431

^*^p < 0.01.

^**^p < 0.001.

### 3.2. Structural equation model

**Figure 2:**
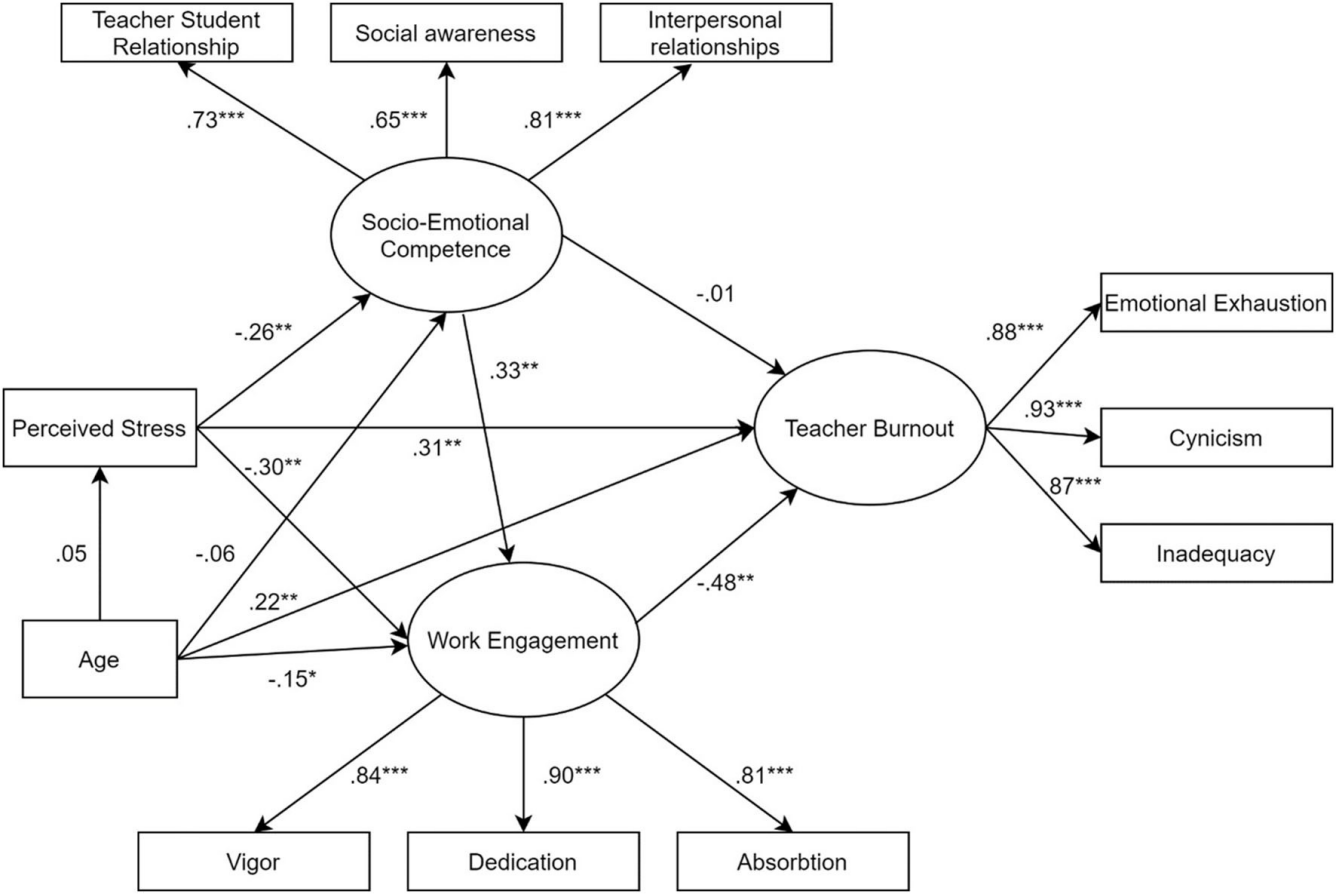
Structural equation modeling of the model of perceived stress, teacher burnout, work engagement and socio-emotional competence. Standardized direct effects are reported. *p < 0.05, **p < 0.01, ***p < 0.001.

The conceptual model revealed (see Figure 2) that there was a good fit between the empirical data and the hypothesized paths between latent variables. All fit indexes supported the model's full acceptance: χ^2^(28) = 78.1 (*p* < 0.001), GFI = 0.941, TLI = 0.945, CFI = 0.964, RMSEA = 0.080, and SRMR = 0.052. The total, direct, and indirect standardized effects are shown in Table 2. The interaction of three variables is referred to as indirect effects, whereas direct effects are the impact of a single determinant on a single target variable.

**Table 2:** Summary of standardized total, direct and indirect effects: structural paths between perceived stress, socio-emotional competence, work engagement, and teacher burnout scores.

**From**	**To**	**Total effect**				**Direct effect**				**Indirect effect**			
		β	***p*** **-value**	**95% CI LB**	**95% CI UB**	β	***p*** **-value**	**95% CI LB**	**95% CI UB**	β	***p*** **-value**	**95% CI LB**	**95% CI UB**
Age	Perceived stress	0.052	0.334	−0.025	0.099	0.052	0.334	−0.025	0.099	–	–	–	–
Age	Teacher burnout	0.330^**^	0.004	0.035	0.078	0.223^**^	0.004	0.022	0.054	0.107^*^	0.013	0.005	0.033
Age	Work engagement	−0.189^**^	0.005	−0.049	−0.013	−0.149^*^	0.015	−0.041	−0.009	−0.040	0.310	−0.017	0.003
Age	Socio-emotional competence	−0.073	0.446	−0.083	0.021	−0.059	0.510	−0.079	0.022	−0.014	0.338	−0.013	0.003
Perceived stress	Teacher burnout	0.500^**^	0.004	0.098	0.170	0.311^**^	0.004	0.055	0.112	0.189^**^	0.004	0.033	0.070
Perceived stress	Work engagement	−0.391^**^	0.004	−0.130	−0.073	−0.303^**^	0.004	−0.106	−0.052	−0.088^**^	0.007	−0.037	−0.007
Perceived stress	Socio-emotional competence	−0.264^**^	0.008	−0.202	−0.062	−0.264^**^	0.008	−0.202	−0.062	–	–	–	–
Socio-emotional competence	Teacher burnout	−0.167^*^	0.014	−0.141	−0.031	−0.007	0.929	−0.060	0.049	−0.160^**^	0.004	−0.125	−0.041
Socio-emotional competence	Work engagement	0.334^**^	0.004	0.089	0.241	0.334^**^	0.004	0.089	0.241	–	–	–	–
Work engagement	Teacher burnout	−0.480^**^	0.004	−0.667	−0.349	−0.480^**^	0.004	−0.667	−0.349	–	–	–	–

^*^p < 0.05.

^**^p < 0.01.

CI, confidence interval; LB, lower bound; UB, upper bound.

Beginning at the bottom and moving upward, the perceived stress variable had a large, positive, and statistically significant total effect on burnout (β = 0.50, *p* < 0.001). This effect was the result of the combination of the direct effect (β = 0.31, *p* < 0.001) and the indirect effect (β = 0.19, *p* < 0.001) via socio-emotional competence and work engagement. In point of fact, an examination of the direct paths revealed a negative effect in the association of perceived stress with both socio-emotional competence (β = −0.26, *p* < 0.001) and work engagement (β = −0.30, *p* < 0.001). This finding suggests that increasing levels of perceived stress correspond to an increase in burnout and a decrease in both socio-emotional competence and work engagement. The total effect of socio-emotional competence on burnout was negative and statistically significant (β = −0.17, *p* < 0.001), however a closer look at the paths revealed that the direct effect was not statistically significant but that the indirect effect was statistically significant (β = −0.16, *p* < 0.001), meaning that socio-emotional competence can help in reducing levels of burnout only via its effect on work engagement. Also, work engagement showed a negative and statistically significant effect on burnout (β = −0.48, *p* < 0.001). Finally, age was positively associated with burnout (β =0.33, *p* < 0.05) and negatively associated with work engagement (β = −0.19, *p* < 0.001).

## 4. Discussion

The aim of this study was to innovatively investigate direct and indirect effects of teachers' perceived stress on burnout considering also the effects of socio-emotional competence and work engagement, when controlling for age using a unique comprehensive model. The section below discusses the findings of both the correlation analysis and the structural equation model, which elucidate the direct and indirect effects that govern the cumulative network of relationships among these variables.

The correlation findings aligned with previous research that has documented significant associations between these variables. Specifically, consistent with the literature, teachers with higher levels of perceived stress also had higher levels of burnout (Rey et al., 2016; Bottiani et al., 2019). Furthermore, teachers with higher social-emotional competences tended to be more engaged in their work and to experience lower levels of burnout (Brackett et al., 2010; D'Amico et al., 2020; Geraci et al., 2023), as well as lower levels of stress (Messineo and Tosto, 2023). These associations highlighted their importance in understanding teacher wellbeing and job performance (Lester et al., 2020).

With regard to the results of the structural equation model, as expected (H1) we found a positive and significant direct effect of perceived stress on teacher burnout. This is in line with numerous studies that have emphasized the detrimental impact of high levels of stress on individuals' wellbeing and job satisfaction (e.g., Demerouti et al., 2001; Maslach and Leiter, 2008).

The negative effect of perceived stress on both socio-emotional competence and work engagement reinforced the notion that stress can impede individuals' ability to effectively manage their emotions and engage in their work tasks (Salovey and Mayer, 1990; Bakker and Demerouti, 2017). In fact, high levels of perceived stress can reduce teachers' ability to accurately recognize and regulate their own emotions making difficult to effectively manage their emotional responses and actively being engaged in their job. These findings emphasize the importance of promoting socio-emotional competencies and creating conducive work environments that foster engagement (Cavioni et al., 2023), as they can act as protective factors against the negative impact of stress.

Confirming our hypothesis (H2) we found a negative effect of work engagement on burnout. Maricuţoiu et al. (2017) reported negative effects between these variables, even if the path was stronger from burnout to work engagement. The effect we found highlighted the significance of developing strategies to prevent burnout by enhancing work engagement, such as implementing job resources, fostering positive work relationships, promoting mutual support and providing opportunities for professional development (Hakanen et al., 2006; Bakker and Demerouti, 2014).

We found that even socio-emotional competence had an effect on work engagement, as expected (H3). This finding aligns with previous research indicating that individuals with higher socio-emotional competence are more likely to be engaged in their work (Mérida-López et al., 2022), and emphasizes the importance of developing and promoting socio-emotional skills among teachers to enhance their job satisfaction, performance, and overall wellbeing. Previous studies have documented the protective role of socio-emotional competence, especially emotion regulation skills, in reducing the negative effect of stress and anxiety, which are feelings that are frequently experienced in the teaching profession. Also, high emotional competence helped teachers facing the negative feelings (fear, loss, etc.) experienced in difficult and challenging situations—such as the COVID-19 pandemic. In this way, high competence helped to maintain good levels of work engagement and reduced the risk of burnout (Geraci et al., 2023).

Interestingly, social-emotional competences seemed to not have a direct effect on burnout, as their effect passes through teachers' work engagement. This meant that enhancing teachers' personal skills would not be effective unless vigor, absorption, and dedication to the professional activities were reinforced as well. Thus, the model we tested, which innovatively investigates the effect of stress on burnout by simultaneously considering the contribution of social-emotional skills and work engagement, makes it possible to state that training interventions to prevent burnout at school should simultaneously enhance teachers' social-emotional competences (e.g., emotion regulation) and support positive work-related mental states.

With regard to age, we found that it had a direct positive effect on burnout and a negative effect on work-engagement. This implied that older teachers, tend to lose motivation in their professional life and become more at risk of burnout symptoms, such as emotional exhaustion. This was consistent with studies that have suggested a higher risk of burnout among older employees (e.g., Schaufeli et al., 2009; Demerouti et al., 2010) and research highlighting potential declines in engagement as individuals progress in their careers (Zacher, 2015). This outcome also implies that age-related factors, such as increased work demands, reduced resilience, or diminished job resources, may contribute to higher burnout levels. These findings underscore the importance of tailoring interventions and support systems to address the specific needs and challenges faced by different age groups of teachers. Morevover, it is important to invest in early teacher training, starting with pre-service teachers, so that they can acquire valuable resources that will help them reduce the negative effect of prolonged stress on their wellbeing.

Overall, the structural equation model provided valuable insights into the relationships between perceived stress, socio-emotional competence, work engagement, and burnout. The findings suggest that perceived stress significantly influences burnout levels, with both direct and indirect effects through socio-emotional competence and work engagement. Furthermore, our findings showed that socio-emotional competence and work engagement have distinct roles in reducing burnout, with socio-emotional competence affecting burnout only through its impact on work engagement. The results also highlight the influence of age, showing positive associations with burnout and negative associations with work engagement.

In conclusion, the results of this study contribute to the existing literature by providing a deeper understanding of the associations between perceived stress, burnout, socio-emotional competence, and work engagement in a sample of in-service teachers. The findings emphasize the need for interventions that target not only stress reduction (as in the very recent study by Inácio et al., 2023), but also foster socio-emotional competence, enhance work engagement, and address age-related factors to promote teacher wellbeing and job performance. By incorporating these insights into educational policies and practices, stakeholders can create supportive and sustainable work environments that nurture the professional growth and satisfaction of teachers.

### 4.1. Limitations and future research directions

This investigation, just like many other studies, was subject to a number of limitations that ought to be taken into consideration. The fact that the research was designed using a cross-sectional approach is the first. As a result, it was impossible to determine with “absolute” confidence what factors led to the observed outcomes. In order to address this issue, structural equation modeling was utilized. These models made it possible to analyze both the direct and indirect impacts of variables inside a single cohesive structure. However, it was essential to keep in mind that the findings should be discussed in terms of probabilistic causality, not deterministic causality (Glymour et al., 2016).

A second limitation of the study was that the sample of teachers who participated was not completely representative of the teaching population. In point of fact, the participants were recruited using a sampling method that did not rely on probability, since they were chosen from the general population solely on the basis of their willingness to take part. Because of this, the findings can only be generalized to the population of teachers that was being studied to a limited extent. In addition, most participants were female, which makes the sample unbalanced. However, it should be emphasized that the ratio of male to female participants is comparable to that which has been observed in the real population of Italian teachers.

Moreover, the cross-sectional nature of the study does not allow us to interpret our results in terms of cause-effect. In the future, therefore, it will be useful to conduct intervention-studies to validate training programs aimed at strengthening teachers' social-emotional and work-engagement skills in order to prevent the impact of perceived stress on burnout risk.

In conclusion, it is essential to bear these limits in mind when interpreting the results of this study. Rather than viewing the findings as a comprehensive and unquestionable portrayal, they are an open contribution to the knowledge of the phenomenon that was investigated. However, the use of structural equation models, the robustness of the statistical output, and the fact that the male-to-female ratio was aligned with the population of Italian teachers are strengths of the study that clearly fortify its reliability and validity.

## Data availability statement

The raw data supporting the conclusions of this article will be made available by the authors, without undue reservation.

## Ethics statement

The study received the approval by the Ethics Committee of the University of Milano-Bicocca and it was conducted in accordance with the local legislation and institutional requirements. The participants provided their written informed consent to participate in this study.

## Author contributions

VO: Conceptualization, Funding acquisition, Investigation, Methodology, Supervision, Writing—original draft, Writing—review & editing. EC: Conceptualization, Data curation, Methodology, Writing—original draft. VC: Conceptualization, Data curation, Methodology, Writing—original draft. EF: Conceptualization, Writing—original draft. AP: Conceptualization, Formal analysis, Writing—original draft.

## References

[B1] Bakker A. B. Bal M. P. (2010). Weekly work engagement and performance: a study among starting teachers. J. Occup. Organ. Psychol. 83, 189–206. 10.1348/096317909X402596

[B2] Bakker A. B. Demerouti E. (2014). “Job demands-resources theory,” in Work and Wellbeing, eds P. Y. Chen, and C. L. Cooper (Hoboken, NJ: Wiley Blackwell), 37–64. 10.1002/9781118539415.wbwell019

[B3] Bakker A. B. Demerouti E. (2017). Job demands–resources theory: taking stock and looking forward. J. Occup. Health Psychol. 22, 273–285. 10.1037/ocp0000056 27732008

[B4] Bakker A. B. Demerouti E. Verbeke W. (2004). Using the job demands-resources model to predict burnout and performance. Hum. Resour. Manage. 43, 83–104. 10.1002/hrm.20004

[B5] Bianchi R. Schonfeld I. S. (2016). Burnout is associated with a depressive cognitive style. Pers. Indiv. Differ. 100, 1–5. 10.1016/j.paid.2016.01.008

[B6] Bianchi R. Schonfeld I. S. Laurent E. (2014). Is burnout a depressive disorder? A reexamination with special focus on atypical depression. Int. J. Stress Manage. 21, 307–324. 10.1037/a0037906

[B7] Bottiani J. H. Duran C. A. K. Pas E. T. Bradshaw C. P. (2019). Teacher stress and burnout in urban middle schools: associations with job demands, resources, and effective classroom practices. J. Sch. Psychol. 77, 36–51. 10.1016/j.jsp.2019.10.002 31837727

[B8] Brackett M. A. Palomera R. Mojsa-Kaja J. Reyes M. R. Salovey P. (2010). Emotion-regulation ability, burnout, and job satisfaction among British secondary-school teachers. Psychol. Schools 47, 410–417. 10.1002/pits.20478

[B9] Brackett M. A. Reyes M. R. Rivers S. E. Elbertson N. A. Salovey P. (2012). Assessing teachers' beliefs about social and emotional learning. J. Psychoeduc. Assess. 30, 219–236. 10.1177/0734282911424879

[B10] Capone V. Joshanloo M. Sang-Ah Park M. (2019). Burnout, depression, efficacy beliefs, and work-related variables among school teachers. Int. J. Educ. Res. 95, 97–108. 10.1016/j.ijer.2019.02.001

[B11] Carroll A. Forrest K. Sanders-O'Connor E. Flynn L. Bower J. M. Fynes-Clinton S.. (2022). Teacher stress and burnout in Australia: examining the role of intrapersonal and environmental factors. Soc. Psychol. Educ. 25, 441–469. 10.1007/s11218-022-09686-7 35233183 PMC8874312

[B12] Cavioni V. Grazzani V. Ornaghi V. Agliati A. Gandellini S. Cefai C.. (2023). A multi-component curriculum to promote teachers' mental health: findings from the PROMEHS program. Int. J. Emot. Educ. 15, 34–52. 10.56300/KFNZ2526

[B13] Cece V. Guillet-Descas E. Lentillon-Kaestner V. (2022). The longitudinal trajectories of teacher burnout and vigour across the scholar year: the predictive role of emotional intelligence. Psychol. Sch. 59, 589–606. 10.1002/pits.22633

[B14] Cefai C. Camilleri L. Bartolo P. Grazzani I. Cavioni V Conte E.. (2022). The effectiveness of a school-based, universal mental health programme in six European countries. Front. Psychol. 13, 925614. 10.3389/fpsyg.2022.925614 36003110 PMC9393716

[B15] Chang M. L. (2009). An appraisal perspective of teacher burnout: examining the emotional work of teachers. Educ. Psychol. Rev. 21, 193–218. 10.1007/s10648-009-9106-y

[B16] Cohen S. Williamson G. (1988). “Perceived stress in a probability sample of the United States,” in The Social Psychology of Health, eds S. Spacapan, and S. Oskamp (Newbury Park, CA: Sage), 31–68.

[B17] Conte E. Ornaghi V. Grazzani I. Pepe A. Cavioni V. (2019). Emotion knowledge, theory of mind, and language in young children: testing a comprehensive conceptual model. Front. Psychol. 10, 21–44. 10.3389/fpsyg.2019.02144 31607984 PMC6761293

[B18] Cooper C. Travers C. (2012). Teachers Under Pressure: Stress in the teaching Profession. New York, NY: Routledge. 10.4324/9780203059975

[B19] D'Amico A. Geraci A. Tarantino C. (2020). The relationship between perceived emotional intelligence, work engagement, job satisfaction, and burnout in Italian school teachers: an exploratory study. Psychol. Topics 29, 63–84. 10.31820/pt.29.1.4

[B20] Demerouti E. Bakker A. B. Nachreiner F. Schaufeli W. B. (2001). The job demands-resources model of burnout. J. App. Psychol. 86, 499–512. 10.1037/0021-9010.86.3.499 11419809

[B21] Demerouti E. Mostert K. Bakker A. B. (2010). Burnout and work engagement: a thorough investigation of the independency of both constructs. J. Occup. Health Psychol. 15, 209–222. 10.1037/a0019408 20604629

[B22] Emerson R. W. (2015). Convenience sampling, random sampling, and snowball sampling: how does sampling affect the validity of research? J. Visual Impair. Blin. 109, 164–168. 10.1177/0145482X1510900215

[B23] Fiorilli C. De Stasio S. Di Chiacchio C. Pepe A. Salmela-Aro K. (2017). School burnout, depressive symptoms and engagement: their combined effect on student achievement. Int. J. Educ: Res. 84, 1–12. 10.1016/j.ijer.2017.04.001

[B24] Froehlich D. E. Morinaj J. Guias D. Hobusch U. (2022). Newly qualified teachers' well-being during the COVID-19 pandemic: testing a social support intervention through design-based research. Front. Psychol. 13, 873797. 10.3389/fpsyg.2022.873797 35747674 PMC9209762

[B25] Geraci A. Di Domenico L. Inguglia C. D'Amico A. (2023). Teachers' emotional intelligence, burnout, work engagement, and self-efficacy during COVID-19 lockdown. Behav. Sci. 13, 296. 10.3390/bs13040296 37102810 PMC10135634

[B26] Glymour M. Pearl J. Jewell N. P. (2016). Causal *Inference* in *Statistics: A Primer*. Hoboken, NJ: John Wiley and Sons.

[B27] Hakanen J. J. Bakker A. B. Schaufeli W. B. (2006). Burnout and work engagement among teachers. J. School Psychol. 43, 495–513. 10.1016/j.jsp.2005.11.001

[B28] Holeyannavar P. G. Itagi S. K. (2012). Stress and emotional competence of primary school teachers. J. Psychol. 3, 29–38. 10.1080/09764224.2012.11885475

[B29] Inácio S. Pitacho L. Moreira A. Tomás C. (2023). Occupational stress and sleep smong teachers: what is the effective-ness of a programme to reduce occupational stress using mindfulness? Sch. J. Psychol. Behav. Sci. 7, 856–866. 10.32474/SJPBS.2023.07.000262

[B30] Jennings P. A. (2011). “Promoting teachers' social and emotional competencies to support performance and reduce burnout,” in Breaking the Mold of Preservice and Inservice Teacher Education: Innovative and Successful Practices for the Twenty-first Century, eds A. Cohan, and A. Honigsfeld (Lanham, MD: Rowman and Littlefield), 133–143.

[B31] Jennings P. A. Greenberg M. T. (2009). The prosocial classroom: teacher social and emotional competence in relation to student and classroom outcomes. Rev. Educ. Res. 79, 491–525. 10.3102/0034654308325693

[B32] Kenny D. A. Kaniskan B. McCoach D. B. (2015). The performance of RMSEA in models with small degrees of freedom. Sociol. Methods Res. 44, 486–507. 10.1177/0049124114543236

[B33] Khan A. Ud Din S. Anwar M. (2019). Sources and adverse effects of burnout among academic staff: a systematic review. City Univ. Res. J. 9, 350–362.

[B34] Kyriacou C. (2011). “Teacher stress: from prevalence to resilience,” in andbook of Stress in the Occupations, eds J. Langan-Fox, and C. L. Cooper (London: Edward Elgar Publishing), 161–173. 10.4337/9780857931153.00026

[B35] Lester L. Cefai C. Cavioni V. Cross D. Barnes A. (2020). A whole-school approach to promoting staff wellbeing. Aust. J. Teach. Educ. 45, 1–22. 10.14221/ajte.2020v45n2.1

[B36] Levante A. Petrocchi S. Bianco F. Castelli I. Lecciso F. (2023). Teachers during the COVID-19 era: the mediation role played by mentalizing ability on the relationship between depressive symptoms, anxious trait, and job burnout. Int. J. Env. Res. Public Health 20, 859. 10.3390/ijerph20010859 36613181 PMC9820251

[B37] Li S. Sheng Y. Jing Y. (2022). How social support impact teachers' mental health literacy: a chain mediation model. Front. Psychol. 13, 851332. 10.3389/fpsyg.2022.851332 35369149 PMC8968130

[B38] Lizana P. A. Vega-Fernadez G. Gomez-Bruton A. Leyton B. Lera L. (2021). Impact of the COVID-19 pandemic on teacher quality of life: a longitudinal study from before and during the health crisis. Int. J. Env. Res. Public Health 18, 3764. 10.3390/ijerph18073764 33916544 PMC8038473

[B39] Marcoulides G. A. Schumacker R. E. (2001). New Developments and Techniques In Structural Equation Modeling. London: Psychology Press. 10.4324/9781410601858

[B40] Maricuţoiu L. P. Sulea C. Iancu A. (2017). Work engagement or burnout: which comes first? A meta-analysis of longitudinal evidence. Burn. Res. 5, 35–43. 10.1016/j.burn.2017.05.001

[B41] Marsh H. W. Morin A. J. Parker P. D. Kaur G. (2014). Exploratory structural equation modeling: an integration of the best features of exploratory and confirmatory factor analysis. Annu. Rev. Clin. Psychol. 10, 85–110. 10.1146/annurev-clinpsy-032813-153700 24313568

[B42] Maslach C. Leiter M. P. (2008). Early predictors of job burnout and engagement. J. App. Psychol. 93, 498–512. 10.1037/0021-9010.93.3.498 18457483

[B43] Maslach C. Schaufeli W. B. Leiter M. P. (2001). Job burnout. Annu. Rev. Psychol. 52, 397–422. 10.1146/annurev.psych.52.1.397 11148311

[B44] Mérida-López S. Carvalho V. S. Chambel M. J. Extremera N. (2023). Emotional intelligence and teachers' work engagement: the mediating and moderating role of perceived stress. J. Psychol. 157, 212–226. 10.1080/00223980.2023.2169231 36808906

[B45] Mérida-López S. Extremera N. (2017). Emotional intelligence and teacher burnout: a systematic review. Int. J. Educ. Res. 85, 121–130. 10.1016/j.ijer.2017.07.006

[B46] Mérida-López S. Extremera N. Rey L. (2017). Contributions of work-related stress and emotional intelligence to teacher engagement: additive and interactive effects. Int. J. Env. Res. Public Health 14, 1156. 10.3390/ijerph14101156 28961218 PMC5664657

[B47] Mérida-López S. Quintana-Orts C. Rey L. Extremera N. (2022). Teachers' subjective happiness: testing the importance of emotional intelligence facets beyond perceived stress. Psychol. Res. Behav. Manag. 15, 317–326. 10.2147/PRBM.S350191 35210880 PMC8859289

[B48] Messineo L. Tosto C. (2023). Perceived stress and affective experience in Italian teachers during the COVID-19 pandemic: correlation with coping and emotion regulation strategies. Eur. J. Psychol. Educ. 38, 1271–1293. 10.1007/s10212-022-00661-6 PMC9734932 40479454

[B49] Montgomery C. Rupp A. A. (2005). A meta-analysis for exploring the diverse causes and effects of stress in teachers. Can. J. Educ. 28, 458–486. 10.2307/4126479

[B50] Nalipay M. J. N. King R. B. Haw J. Y. Mordeno I. G. Dela Rosa E. D. (2021). Teachers who believe that emotions are changeable are more positive and engaged: the role of emotion mindset among in- and preservice teachers. Learn. Indiv. Differ. 92, 102050. 10.1016/j.lindif.2021.102050

[B51] Ornaghi V. Pepe A. Agliati A. Grazzani I. (2019). The contribution of emotion knowledge, language ability, and maternal emotion socialization style to explaining toddlers' emotion regulation. Soc. Dev. 28, 581–598. 10.1111/sode.12351

[B52] Pellerone M. (2021). Self-perceived instructional competence, self-efficacy and burnout during the COVID-19 pandemic: a study of a group of Italian school teachers. Eur. J. Investig. Health Psychol. Educ. 11, 496–512. 10.3390/ejihpe11020035 34708818 PMC8314360

[B53] Pepe A. Ornaghi V. Belacchi C. Farina E. (2023). Alexithymia as a risk factor for social indifference: a quantitative study with a large sample of female adolescents. Sch. Ment. Health 15, 1–12. 10.1007/s12310-023-09568-z

[B54] Peterson C. Maier S. F. Seligman M. E. P. (1993). Learned Helplessness: A Theory for the Age of Personal Control. Oxford: Oxford University Press.

[B55] Pisanti R. Paplomatas A. Bertini M. (2008). Measuring the positive dimensions among health care workers: a contribution to the Italian validation of the UWES-Utrecht Work Engagement Scale. G. Ital. Med. Lav. Ergon. 30, 111–119. 18700486

[B56] Preacher K. J. Zhang Z. Zyphur M. J. (2011). Alternative methods for assessing mediation in multilevel data: the advantages of multilevel SEM. Struct. Equ. Modelling 18, 161–182. 10.1080/10705511.2011.557329

[B57] Rey L. Extremera N. Pena M. (2016). Emotional competence relating to perceived stress and burnout in Spanish teachers: a mediator model. PeerJ 4, e2087. 10.7717/peerj.2087 27280077 PMC4893324

[B58] Ripplinger J. Sullivan J. (2008). Does choice in model selection affect maximum likelihood analysis? Systematic Biol. 57, 76–85. 10.1080/10635150801898920 18275003

[B59] Salmela-Aro K. Rantanen J. Hyvönen K. Tilleman K. Feldt T. (2011). Bergen burnout inventory: reliability and validity among Finnish and Estonian managers. Int. Arch. Occ. Environ Health 84, 635–645. 10.1007/s00420-010-0594-3 21082191

[B60] Salovey P. Mayer J. D. (1990). Emotional intelligence. Imagin. Cogn. Pers. 9, 185–211. 10.2190/DUGG-P24E-52WK-6CDG

[B61] Schaufeli W. B. Bakker A. B. Salanova M. (2006). The measurement of work engagement with a short questionnaire: a cross-national study. Educ. Psychol. Meas. 66, 701–716. 10.1177/0013164405282471

[B62] Schaufeli W. B. Bakker A. B. Van Rhenen W. (2009). How changes in job demands and resources predict burnout, work engagement, and sickness absenteeism. J. Organ. Behav. 30, 893–917. 10.1002/job.595

[B63] Schaufeli W. B. Buunk B. P. (2004). “Burnout: an overview of 25 years of research and theorizing,” in The Handbook of Work and Health Psychology, 2nd ed., eds M. J. Schabracq, J. A. M. Winnubst, and C. L. Cooper (Hoboken, NJ: Wiley), 383–425. 10.1002/0470013400.ch19

[B64] Schaufeli W. B. Martínez I. Marques-Pinto A. Salanova M. Bakker A. B. (2002). Burnout and engagement in university students: a cross national study. J. Cross Cult. Psychol. 33, 464–481. 10.1177/0022022102033005003

[B65] Shi D. Maydeu-Olivares A. (2020). The effect of estimation methods on SEM fit indices. Educ. Psychol. Meas. 80, 421–445. 10.1177/0013164419885164 32425213 PMC7221491

[B66] Skaalvik E. M. Skaalvik S. (2016). Teacher stress and teacher self-efficacy as predictors of engagement, emotional exhaustion, and motivation to leave the teaching profession. Creat. Educ. 7, 1785–1799. 10.4236/ce.2016.713182

[B67] Tavolacci M. P. Ladner J. Grigioni S. Richard L. Villet H. Dechelotte P.. (2013). Prevalence and association of perceived stress, substance use and behavioral addictions: a cross-sectional study among university students in France, 2009–2011. BMC Public Health 13, 724. 10.1186/1471-2458-13-724 23919651 PMC3750571

[B68] Thakkar J. J. (2020). Structural Equation Modelling*: Application for Research and Practice (with AMOS and R)*. New York, NY: Springer. 10.1007/978-981-15-3793-6

[B69] Tom K. M. (2012). Measurement of Teachers' Social-Emotional Competence: Development of the Social-Emotional Competence Teacher Rating Scale [Doctoral dissertation]. Available online at: https://scholarsbank.uoregon.edu/xmlui/bitstream/handle/1794/12351/Tom_oregon_0171A_10250.pdf?sequence=1andisAllowed=y (accessed October 16, 2023).

[B70] Vargas Rubilar N. Oros L. B. (2021). Stress and burnout in teachers during times of pandemic. Front. Psychol. 12, 756007. 10.3389/fpsyg.2021.756007 34899498 PMC8660691

[B71] Ximénez C. Maydeu-Olivares A. Shi D. Revuelta J. (2022). Assessing cutoff values of SEM fit indices: advantages of the unbiased SRMR index and its cutoff criterion based on communality. Struct. Equ. Modeling 29, 368–380. 10.1080/10705511.2021.1992596

[B72] Zacher H. (2015). Daily manifestations of career adaptability: relationships with job and career outcomes. J. Vocat. Behav. 91, 76–86. 10.1016/j.jvb.2015.09.003

